# Optimization of Printing Parameters to Maximize the Mechanical Properties of 3D-Printed PETG-Based Parts

**DOI:** 10.3390/polym14132564

**Published:** 2022-06-24

**Authors:** Sara Valvez, Abilio P. Silva, Paulo N. B. Reis

**Affiliations:** 1 Department of Electromechanical Engineering, CMAST, University of Beira Interior, 6201-001 Covilhã, Portugal; sara.valvez@ubi.pt (S.V.); abilio@ubi.pt (A.P.S.); 2 Department of Mechanical Engineering, CEMMPRE, University of Coimbra, 3030-194 Coimbra, Portugal

**Keywords:** additive manufacturing, FFF, design of experiments, printing parameters, mechanical properties, thermal conductivity, mechanical testing

## Abstract

Fused filament fabrication (FFF) is the most popular additive manufacturing method, which allows the production of highly complex three-dimensional parts with minimal material waste. On the other hand, polyethylene terephthalate glycol (PETG) has been used to replace traditional polymers for 3D printing due to its chemical resistance and mechanical performance, among other benefits. However, when fibres are added, these PETG-based composites can be suitable for many different applications. Nevertheless, to guarantee their good performance in-service in these applications, and even extend to new ones, it is necessary for their mechanical properties to be maximized. Therefore, this study intends to optimize the printing parameters (nozzle temperature, printing speed, layer height and filling) in order to maximize the mechanical properties of printed PETG, PETG+CF (carbon fibre-reinforced PETG composites) and PETG+KF (aramid fibre-reinforced PETG composites). The Taguchi method was used for the experimental procedure design, and the specimens were produced according to the L16 orthogonal array. Finally, an analysis of variance (ANOVA) was performed, with a 95% confidence interval, to analyse the effect of the printing parameters on the bending properties. It was possible to conclude that the best bending properties for PETG, PETG+CF and PETG+KF were obtained for extrusion temperatures of 265 °C, 195 °C and 265 °C, printing speeds of 20, 60 and 20 mm/s, layer heights of 0.4, 0.53 and 0.35 mm and an infill density of 100% for the three materials, respectively.

## 1. Introduction

Additive manufacturing (AM) methods are a group of techniques capable of producing three-dimensional parts directly from CAD data [1], and they have the potential for use in multiple applications in the defence, aircraft, medical and automobile industries. These technologies are still under the development stage due to the high cost of equipment and materials used [2], but they are very promising because they involve lower cost processes, require less manufacturing time and provide higher flexibility [3].

According to ASTM (the American Society for Testing and Materials), AM methods are divided into seven groups: powder bed fusion (PBF), binder jetting, material jetting, sheet lamination, direct energy deposition (DED), vat photopolymerization and material extrusion [4]. Among all of these, the literature reports FFF (fused filament fabrication) as being the most promising and widely used technology that is included in the material extrusion group. It is often confused with fused deposition modelling (FDM), a trademark created by Stratasys’ co-founder Scott Crump in 1988 and commercially available in the late 1990s [5]. In this case, a continuous filament of thermoplastic polymer is heated at the nozzle to a semi-liquid condition, and then extruded onto the platform or on top of the pre-printed layers. The thermoplasticity of the raw material is critical for this printing technique because it allows the filaments to fuse during printing and solidify at room temperature after printing [6,7]. The main advantages of this technique are its simplicity, fast process and moderate cost. However, several drawbacks, such as poor mechanical properties, surface quality and appearance, as well as a limited number of raw materials, are reported in the literature [7,8].

In terms of mechanical properties, for example, these can be improved by post-processing treatments [9], the addition of reinforcements to the polymeric matrix [10] and optimization of the printing parameters [11]. For the first case, annealing is a post-processing heat treatment commonly used to improve the mechanical properties and the superficial aspect [12]. Hong et al. [13], for example, reported benefits in terms of bending and compressive strength for PLA of around 58.3% and 39.8%, respectively, after heat treatment. In this study, the highest bending strength was obtained at 130 °C for 300 s, while the highest compressive strength was observed at 140 °C for 600 s. In this case, the heat treatment process reduces the debonding zones between the filaments; however, depending on the direction of the external load, the mechanical strength can be affected by slippage of the filaments. Bhandari et al. [14] obtained improvements around two and three times higher, respectively, for the tensile strength of PLA and PETG composites reinforced with short carbon fibres after annealing. Kumar et al. [15] analysed the annealing effect on hardness, tensile strength, impact strength and bending strength, and they found benefits of around 21%, 25%, 23% and 18%, respectively, for PETG composites.

Regarding the addition of reinforcements to the polymeric matrix, Kasmi et al. [16] investigated the benefits achieved with continuous carbon fibres in PETG-based composites, and found improvements of 9 and 17 times higher, respectively, for the ultimate tensile strength and tensile modulus compared to the neat PETG. In a similar study, Jiang et al. [17] found improvements of 313.2% and 48.2%, respectively, for the tensile modulus and ultimate tensile strength. In terms of tensile strain, they obtained values higher than 25% for neat PETG (exceeded the capacity of the extensometer), which makes the comparison with the filled samples unfeasible. However, according to the authors, the addition of carbon fibres reduces the ductility of the samples by more than 50% in most cases. On the other hand, Ferreira et al. [18] analysed the effect of short carbon fibres (20 wt.%) as reinforcement of a PETG polymer and found improvements of 191.38% and 5.14%, respectively, for the bending modulus and strength, while the tensile strength decreased by 28.1% but the tensile modulus increased by 70.1%. In a similar study, Mansour et al. [19] observed a decrease of 66% in terms of compressive strain, while the compressive modulus and hardness increased by 30% and 27%, respectively, compared to the neat polymer. In terms of compressive strength, Valvez et al. [20] obtained values 9.9% and 68.7% lower when carbon and aramid fibres were added, respectively, to the PETG polymer. The same behaviour was observed for compressive displacement but with values of 20.4% and 46.3%, respectively. On the other hand, the compressive modulus increased by 12.4% when carbon fibres were added to the PETG matrix and decreased by 39.6% for aramid fibres.

All these possibilities to increase the mechanical properties of 3D-printed parts are very efficient, but the most important step is related to the optimization of printing parameters. In fact, this optimization allows a further improvement of the mechanical performance through the techniques described above. Therefore, it is not surprising that the literature reports it as the first, and most important, step towards improving the mechanical properties of FFF printed parts. From all the printing parameters, the literature suggests that the most important are layer thickness, air gaps (in the same layer or between layers), infill density, temperature and printing speed [6,7]. However, due to the complex interaction between parameters, they must be analysed together (as a group) [21]. For this purpose, several design of experiments (DOE) procedures are available in the open literature; the Taguchi method is one of the most used [22,23,24]. Kumar et al. [25], for example, optimized the printing parameters for PETG composites reinforced with carbon fibres using a Taguchi L9 technique. According to the authors, while printing speed and infill density were responsible for maximizing tensile strength and hardness, layer height and infill density were the determinants for bending strength.

Wang et al. [26] concluded that a smaller layer thickness promotes higher tensile strength because it promotes smaller interlayer gaps and, consequently, fewer air pores in the cross-section. Furthermore, the compressed filament deposition by the nozzle under an adequate extrusion force is responsible for increasing the inter-bonding strength. A significant influence of layer thickness and print orientation on the bonding strength of the printed parts was reported by Kovan et al. [27]. These authors observed that the gaps between filament rasters had a detrimental influence on the adhesion properties. Anitha et al. [28] found that the layer thickness significantly affects the surface roughness, even demonstrating an inverse relationship between the layer thickness and surface roughness. Nancharaiah et al. [29] suggested that the surface roughness can be improved by adopting lower values of layer thickness, which simultaneously decreases the size and volume of the voids between the layers, with the consequent improvement in the mechanical properties.

The air gaps, in the same layer or between layers, play a determining role in the inter-bonding forces between printed rasters because, by definition, this parameter represents the distance (or spaces) between two adjacent chains of deposited filaments [30]. In this context, according to Ahn et al. [31], a negative air gap increases the mechanical performance of the parts, but there is a minimum value due to the excess material that can be accumulated in the nozzle and/or in the printed model itself. Furthermore, Dawoud et al. [32] observed that a negative air gap increases the tensile strength due to the increased density of the printed parts. In this case, it is possible to observe that the rasters slightly overlap and, consequently, a stronger interfacial connection occurs between adjacent rasters. On the other hand, positive air gaps result in weak axial connections due to the creation of neighbouring cylindrical rasters in the plane with a reduced contact area.

Kumar et al. [15] studied the infill density (25%, 50%, 75% and 100%) and the annealing treatment of PETG and PETG composites reinforced with 20 wt.% of carbon fibres. The best mechanical properties were obtained with annealed samples and 100% infill density. Johnson et al. [33] analysed the infill density effect (15%, 30%, 50%, 90%, 100%) on the tensile response of different thermoplastics and concluded that the infill density of 100% was the one that led to the highest tensile strength. Studies developed by Kumar et al. [34] evaluated the infill density effect on the tensile properties of Acrylonitrile Styrene Acrylate (ASA) and concluded that an increase in the infill percentage increases its tensile strength. Similar results were found in another study [35], where for infill densities of 40%, 60% and 80%, the maximum tensile strength was obtained for the infill density of 80%.

The literature also reports that the nozzle temperature influences both the fluidity of the extruded filament and its solidification characteristics, as well as the inter-bonding strength between solidified layers [36]. Yang et al. [37], for example, observed that the nozzle temperature affected the crystallinity and mechanical behaviour of parts produced with PEEK. In this case, higher nozzle temperatures promote higher crystallinity, reaching a difference of around 21% between the extreme values of the analysed temperatures (360 °C to 480 °C). This increase was explained by the fact that higher nozzle temperatures provide more energy to the polymer and, consequently, the sample crystallizes for longer periods of time after being deposited. Increasing the nozzle temperature also increases the tensile strength and elastic modulus, while the elongation decreases, due to higher crystallinity and better adhesion between layers. However, it was noticed that very high temperatures degrade the polymer, while low temperatures are responsible for low forces between printing rasters and, in both cases, the mechanical performance is strongly affected. Ding et al. [38] reported that the bending strength of PEI varies insignificantly up to 390 °C but increases progressively with a nozzle temperature above 390 °C due to a significant decrease in air pores. Studies developed by Berreta et al. [39] revealed that nozzle temperature affects the polymer viscosity. In this context, higher viscosities lead to more complex depositions of the filament layer, to which the printing speed must be adjusted. On the other hand, the addition of carbon nanotubes to the polymer significantly increases the viscosity [40,41]. Therefore, higher nozzle temperatures reduce the viscosity and improve the uniformity of the deposited material. However, despite porosity being unavoidable in both materials (neat polymers and composites), higher weight contents of nano-reinforcements significantly increase the porosity due to the agglomeration of nanoparticles in the melted filament during its passage through the printer’s nozzle before deposition.

As described above, print speed is strongly dependent on viscosity and, consequently, on temperature. In this context, the literature provides several studies where the effect of printing speed on the mechanical performance of 3D-printed parts is analysed [42,43,44,45]. Christiyan et al. [46] observed, for example, that increasing print speed decreases the tensile and bending strength due to the lower inter-bonding strength between the solidified layers. According to Abeykoon et al. [47], the printing speed and nozzle temperature must be evaluated simultaneously in order to optimize the solidification conditions of the material. Wang et al. [48], for example, reported that fibre-reinforced composites have a high viscosity and, therefore, high print speeds are not suitable. Literature suggests that under these conditions speeds higher than 30 mm/s are not adequate [24]. A higher print speed reduces the volume of the extruded material and decreases the printing quality, in addition to causing defects. In this context, the mechanical strength of the stacked macromolecule chain decreases with increasing printing speed. On the other hand, when the printing speed is low, the extruded material has enough time to weld with the surrounding printed component, which improves the mechanical performance of the produced parts. Wang et al. [24] observed that increasing the printing speed decreases the tensile strength and density of the printed parts because the printing layers overlap the previous layers that are not yet completely solidified. This reveals that there is not enough time for the polymer chains to diffuse and crystallize, and the poor bonding action results in stratification. On the other hand, the lower printing speeds reduce the voids due to the polymeric chains having more time and energy to recrystallize and, consequently, the bonds are stronger. Therefore, temperature and time (indirectly printing speed) have a significant effect on bond formation [49].

Therefore, this work intends to optimize the printing parameters to maximize the mechanical properties of PETG and PETG composites reinforced with carbon and aramid fibres. A special focus will be given to the effect of reinforcement type, and the level of the printing parameters, nozzle temperature, printing speed, layer height and filling, will be analysed. For this purpose, the Taguchi method and analysis of variance will be used. Three-point bending tests will be used to validate the theoretical results because, according to Banna et al. [50], they are the most sensitive for this purpose. Furthermore, the literature does not present many results for this loading mode, mainly they are in terms of composites, because it focuses essentially on tensile tests or other deposition angles.

## 2. Materials and Methods

Polyethylene terephthalate glycol (PETG) was used, which is a polyester copolymer that has been used to replace traditional polymers for 3D printing due to its durability, flexibility, high impact resistance, high chemical resistance, low moisture absorption, among other benefits. When this polymer is reinforced with carbon fibres, it becomes stronger, more resilient and has less risk of warping. The applications, in this case, extend to the automotive and aeronautical sectors, among other industrial applications. On the other hand, when reinforced with aramid fibres, applications extend to conditions involving high friction and impact resistance. Therefore, considering the benefits described above, PETG, PETG+CF and PETG+KF were the filaments used in this study. While PETG composites containing fibres were supplied by Nanovia (Louargat, France), neat PETG was supplied by FilTech (Baesweiler, Germany).

**Figure 1:**
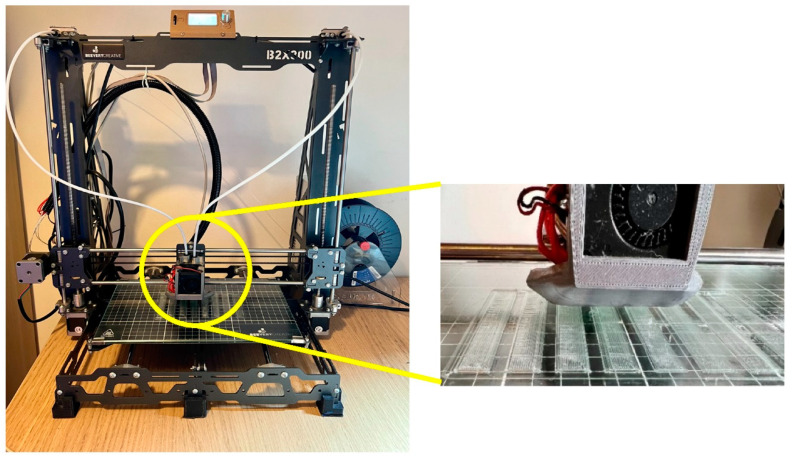
Details of the 3D printer used to produce specimens in a temperature and humidity-controlled room.

In terms of manufacturing process, a B2X300 FDM printer was used to obtain the specimens (see Figure 1). In the FFF technique, a printing head is used to extrude the filament through a 0.6 mm hardened steel nozzle, which is deposited in a pre-defined pattern on a heated platform to create the required flat shape. All samples were printed with a raster angle (direction of deposition) of 0°.

To evaluate the effect of the printing parameters, bending tests were performed according to ASTM D790-17 standard, using a Shimadzu universal testing machine, model Autograph AG-X, with a 5 kN load cell and a span of 64 mm. The dimensions of the samples were 85 × 12.7 × 4 mm and five specimens were tested for each condition at room temperature and with a displacement rate of 2 mm/min. Regardless of the differences between the dimensions of printed and design shape not being studied, the maximum error observed was 5.4%, which would be expected due to low geometric tolerance reported in the literature [51,52]. Therefore, the mechanical properties were obtained with the dimensions measured on the printed parts.

The bending properties were obtained using the following equations:

(1) $$\sigma =\frac{3\;P\;L}{2\;b\;h^{2}}$$

(2) $$E_{f}=\frac{\Delta PL^{3}}{48\Delta uI}\;$$

where *P* is the load, *L* the span length, *b* the width, *h* the thickness of the specimen, *I* the moment of inertia of the cross-section, Δ*P* the load range, Δ*u* the bending displacement range in the mid span for an interval in the linear load-displacement region of the graph. Regarding the bending modulus, it was obtained by linear regression of the load-displacement curves considering the interval in the linear segment with a correlation factor higher than 95%.

**Figure 2:**
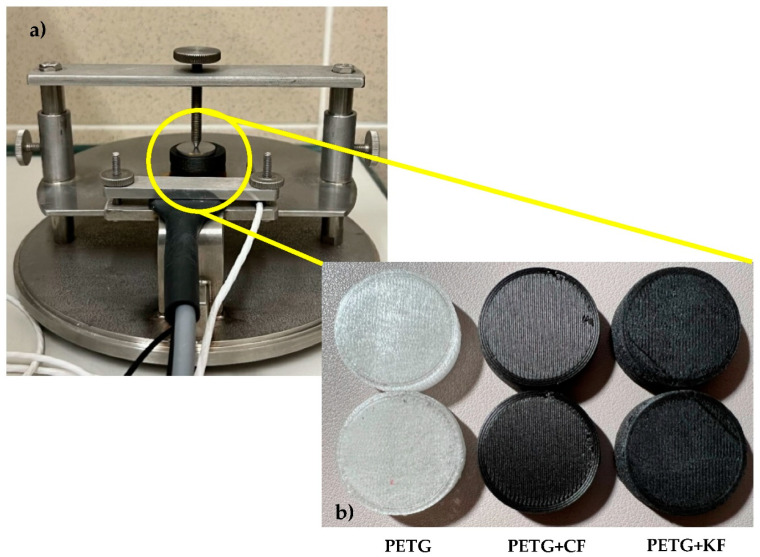
(**a**) Sample and sensor assembly; (**b**) samples used in the thermal conductivity tests.

Finally, a thermal conductivity analysis (k) was performed on a TPS 2500 S thermal constant analyzer (Hot Disk^®^), using the transient flat source method at 20 °C. Measurements were performed using the 5501 sensor (d = 6.4 mm). For each material, 3 cylinders with 30 mm in diameter and 0.8 mm in height were printed and tested. The samples were placed in the respective support with the 5501 sensor between them (see Figure 2).

## 3. Design of Experiment Based on Taguchi Method

The Taguchi method provides a simple and efficient methodology to improve design parameters with the minimum number of well-defined experimental groups. For this reason, the Taguchi method was chosen to select the printing parameters that will maximize the mechanical properties of the parts. However, before choosing an experimental design, it is necessary to define the input parameters from the set of possible values that the 3D printing machine can provide. In this context, four factors were selected for this study, which were represented by letters (A: nozzle temperature, B: speed, C: layer height, D: infill) and, subsequently, defined at four different levels as shown in Table 1, Table 2 and Table 3.

Finally, the Taguchi experimental design to optimize the mechanical properties was determined using the L16 orthogonal array (4^5^) shown in Table 4, Table 5 and Table 6.

In order to maximize the mechanical properties (bending stress, bending modulus and bending strain), five specimens were tested for each trial of the L16 orthogonal array. Response analysis was performed to assess the influence of each factor and was evaluated through the signal-to-noise (S/N) ratio. The obtained S/N results are processed with the larger is better (LB) condition and are calculated by the following equation:

(3) $$S/N_{\mathrm{LB}}=-10{\;\log}_{10}\;\left[\frac{1}{n}\;\overset{n}{\underset{i=1}{\sum }}\frac{1}{R_{i}^{2}}\right]$$

where S/N_LB_ is the signal-to-noise ratio (dB), n is the number of observations under the same experimental conditions and *R_i_* is the response value obtained by the measurement performance.

An analysis of variance (ANOVA) was subsequently performed to develop prediction models and determine the distinguishing factors that influence the bending stress, modulus, and strain. Finally, a desirability function was used to determine the optimum levels of each factor to obtain the best mechanical properties.

## 4. Results

From the tables reported in the previous section, it is possible to obtain the maximum mechanical properties for the different materials studied. In terms of PETG (Table 4), the maximum bending stress was obtained for the combination A5, B2, C1 and D5 (i.e., nozzle temperature of 265 °C, speed of 30 mm/s, layer height of 0.20 mm and infill of 100%) with an average value of 67.07 MPa, while the maximum bending modulus for A3, B1, C3 and D5 (nozzle temperature of 245 °C, speed of 20 mm/s, layer height of 0.30 mm and infill of 100%) with an average value of 1.71 GPa, and the maximum bending strain for A4, B4, C2 and D5 (nozzle temperature of 255 °C, speed of 50 mm/s, layer height of 0.25 mm and infill of 100%) with an average value of 5.76%. For the carbon fibre-reinforced composite (Table 5), these values were 72.75 MPa, 3.52 GPa and 6.83%, respectively, for the combinations A2, B2, C3 and D4 (nozzle temperature of 205 °C, speed of 30 mm/s, layer height of 0.45 mm and infill of 80%); A1, B4, C4 and D4 (nozzle temperature of 195 °C, speed of 50 mm/s, layer height of 0.50 mm and infill of 80%); and A1, B5, C5 and D5 (nozzle temperature of 195 °C, speed of 60 mm/s, layer height of 0.55 mm and infill of 100%). Finally, from Table 6 for the aramid fibre-reinforced composite, the maximum values were 47.35 MPa, 1.5 GPa and 9.18%, respectively, for A5, B2, C1 and D5 (nozzle temperature of 265 °C, speed of 30 mm/s, layer height of 0.35 mm and infill of 100%); A5, B2, C1 and D5 (nozzle temperature of 265 °C, speed of 30 mm/s, layer height of 0.35 mm and infill of 100%); and A5, B1, C5 and D4 (nozzle temperature of 265 °C, speed of 20 mm/s, layer height of 0.55 mm and infill of 80%).

**Figure 3:**
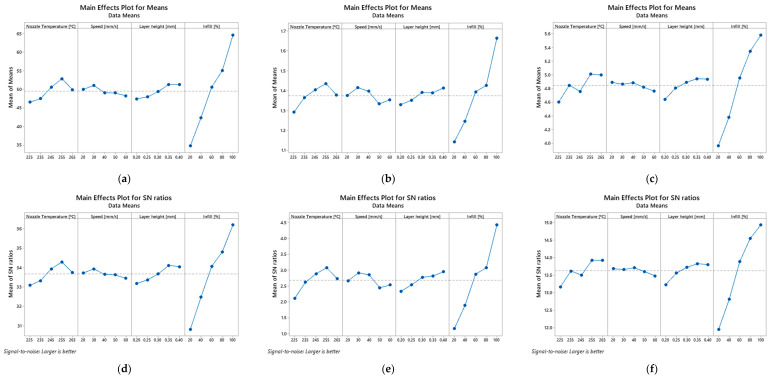
Main effect plot for means of: (**a**) bending stress, (**b**) bending modulus, (**c**) bending strain, and main effect plot for means of S/N ratios of: (**d**) bending stress, (**e**) bending modulus, (**f**) bending strain for PETG samples.

**Figure 4:**
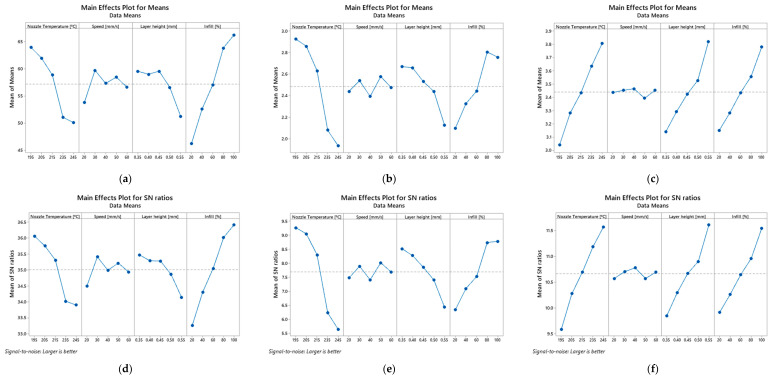
Main effect plot for means of: (**a**) bending stress, (**b**) bending modulus, (**c**) bending strain, and main effect plot for means of S/N ratios of: (**d**) fexural stress, (**e**) bending modulus, (**f**) bending strain for PETG+CF samples.

**Figure 5:**
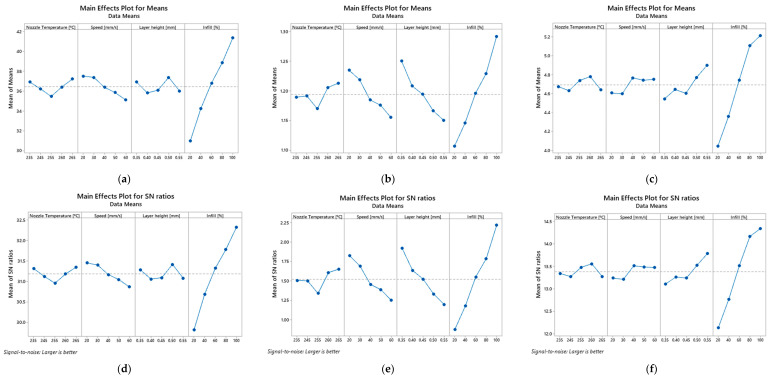
Main effect plot for means of: (**a**) bending stress, (**b**) bending modulus, (**c**) bending strain, and main effect plot for means of S/N ratios of: (**d**) bending stress, (**e**) bending modulus, (**f**) bending strain for PETG+KF samples.

Based on the previous tables, Figure 3, Figure 4 and Figure 5 show the main effect plot for the mean values and S/N ratios on the bending properties for PETG and PETG-based composites. The strongest effect of the controlled factor is determined by the difference between the maximum and minimum values of the S/N ratio. Therefore, for all materials, it can be noticed that the factor related to the infill has the highest influence on the mean values and S/N ratios and, consequently, on the mechanical properties. In this case, an infill equal to 20% minimizes the S/N values for the mechanical properties, while for 100% it maximizes them, denoting a predominant influence. 

Regarding the other factors, they present very small S/N variations and, consequently, a marginal effect on the mechanical properties. There is only one exception to this evidence for composites involving carbon fibres, where the nozzle temperature also has a significant influence on the mean values and S/N ratios. Subsequently, an analysis of variance (ANOVA) was carried out with 95% confidence interval to evaluate the effect of the different printing parameters on the bending properties for the different materials. The results of this study are shown in Table 7, Table 8 and Table 9 for all materials.

**Figure 6:**
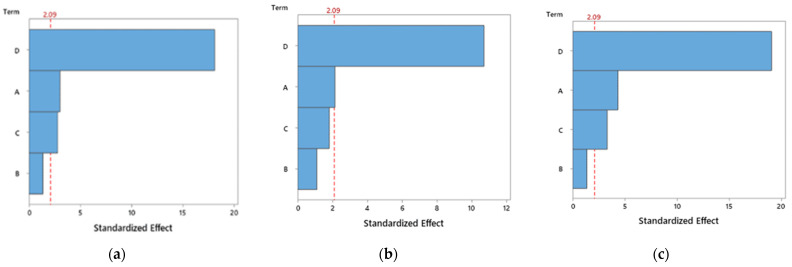
Pareto diagram for PETG and for: (**a**) bending stress; (**b**) bending modulus; (**c**) bending strain.

**Figure 7:**
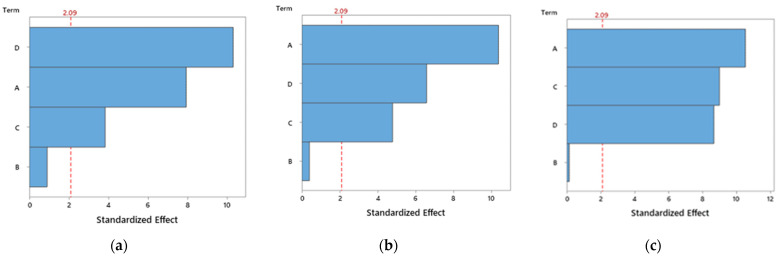
Pareto diagram for PETG+CF and for: (**a**) bending stress; (**b**) bending modulus; (**c**) bending strain.

**Figure 8:**
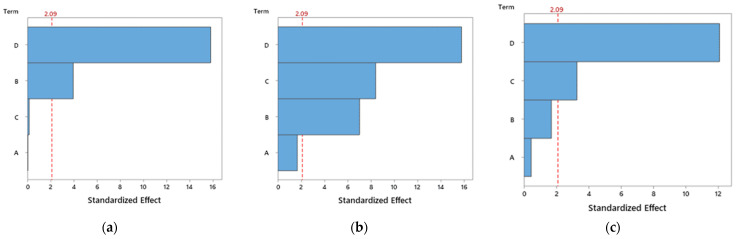
Pareto diagram for PETG+KF and for: (**a**) bending stress; (**b**) bending modulus; (**c**) bending strain.

From this ANOVA analysis for PETG (Table 7), it is possible to conclude that the nozzle temperature, layer height and infill factors have a significant influence on the bending stress and bending strain, because their p-values are less than 0.05. On the other hand, and for the same reasons, only nozzle temperature and infill factors have a significant influence on the bending modulus. However, some F-values are greater than 1, indicating that the variables have variances larger than the error variance, suggesting that these factors have major effects on the responses. Considering the PETG+CF composites (Table 8), all factors have a significant influence on the bending properties, except the speed. However, in relation to PETG+KF composites (Table 9), the factors studied affect each property very specifically. For example, the speed and infill are significant factors for the bending stress, while the speed, layer height and infill are significant factors for the bending modulus, and layer height and infill are significant factors for the bending strain. This analysis is summarized in Figure 6, Figure 7 and Figure 8, which show the Pareto chart of the standardized effects. 

This representation ranks the importance of each factor in descending order based on the severity of the effect, knowing that only the influencing factors cross the reference line (which in the present study is 2.09). Finally, based on the experimental results, a multiple linear regression model was used to correlate the various parameters (A: nozzle temperature, B: velocity, C: layer height and D: infill) with the mechanical properties, according to the following equations:

For PETG

Bending stress = 6.3 + 0.1208 × A − 0.0544×B + 22.23 × C + 0.3614 × D(4)

Bending modulus = 0.334 + 0.00245 × A − 0.00126 × B + 0.414 × C + 0.006114 × D(5)

Bending strain = 0.921 + 0.00956 × A − 0.00299 × B + 1.458 × C + 0.02104 × D(6)

For PETG+CF

Bending stress = 123.25 − 0.3016 × A + 0.0446 × B − 38.23 × C + 0.2571 × D(7)

Bending modulus = 7.821 − 0.02165 × A + 0.00108 × B − 2.617 × C + 0.009 × D(8)

Bending strain = −1.553 + 0.0142 × A − 0.00025 × B + 3.184 × C + 0.00767 × D(9)

For PETG+KF

Bending stress = 31.35 + 0.001 × A − 0.0634 × B − 0.48 × C + 0.12689 × D(10)

Bending modulus = 1.1972 + 0.000632 × A − 0.002023 × B − 0.4839 × C + 0.002273 × D(11)

Bending strain = 2.458 + 0.00152 × A + 0.00433 × B + 1.675 × C + 0.01544 × D(12)

The applied models’ summary for all materials is presented in Table 10, from which it is possible to conclude that the models proposed are statistically significant and reasonably consistent due to the high correlation coefficients obtained.

Finally, an optimal desirability function approach is proposed to simultaneously optimize the multi-response problem with Taguchi’s formulation. A total of 10 solutions were generated to find the best combination that, simultaneously, optimizes the bending stress, modulus, and strain, as shown in Table 11. The optimal point to obtain significant results is found for the desirability equal to 1. Therefore, it is possible to conclude that the best mechanical properties for PETG are obtained for a nozzle temperature of 265 °C, speed of 20 mm/s, layer height of 0.4 mm and infill of 100%. Regarding the PETG+CF composite, these values are 195 °C, 60 mm/s, 0.53 mm and 100%, while for PETG+KF they are 265 °C, 20 mm/s, 0.35 mm and 100%, respectively. To validate the predicted values of Table 11, samples were printed with the combination that proved to be ideal for each material (Table 11).

**Figure 9:**
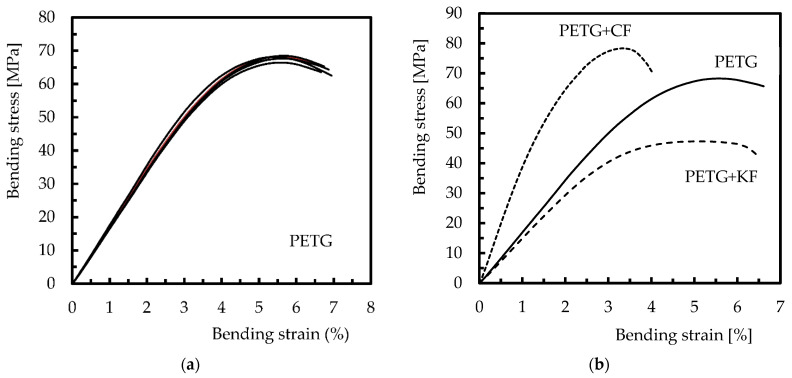
Bending stress–strain curves showing the: (**a**) repeatability of the curves for neat PETG; (**b**) comparison between materials.

Subsequently, the results obtained experimentally were compared with the theoretical/predicted ones. Figure 9 shows the typical bending stress versus strain curves obtained, which are representative of the other conditions. In detail, Figure 9a shows the repeatability of the curves for neat PETG (dispersion similar to that observed for composites), justifying the low dispersion observed in Table 12, while Figure 9b compares the bending properties between materials. Finally, Table 12 highlights the good agreement found between the theoretical and experimental results. This evidence is based on the errors observed between the theoretical and experimental results being always less than 12%, and in some cases both values are almost equal (error of 0.4%).

Finally, to complement this study and evaluate the effect of the fibres on the nozzle temperature parameter, a thermal conductivity analysis was performed, and the results are shown in Table 13. 

## 5. Discussion

From the results presented above, it was possible to conclude that the printing parameters that maximized the bending properties were, for PETG, a nozzle temperature of 265 °C, speed of 20 mm/s, layer height of 0.40 mm and infill of 100%; for PETG+KF a nozzle temperature of 265 °C, speed of 20 mm/s, layer height of 0.35 mm and infill of 100%; and the optimal combination of printing parameters for PETG+CF involved a nozzle temperature of 195 °C, speed of 60 mm/s, layer height of 0.53 mm and infill of 100%. It should be noted that the study aimed to combine the printing parameters to maximize the bending stress, modulus, and strain. In this context, the experimental results led to the following results: bending stress of 66.9 MPa for PETG, 79.2 MPa for PETG+CF and 47.7 MPa for PETG+KF. In terms of the bending modulus, these values were 1.7 GPa, 3.6 GPa and 1.5 GPa, respectively, while for the bending strain they were 5.7%, 3.2% and 5.1%, respectively.

As reported above, literature does not present many results comparable to those obtained in this work because it focuses essentially on tensile tests. However, of those available, Mahesh et al. [53] found for PETG, with a similar raster direction, values of the bending stress and modulus around 56 MPa and 1.5 GPa, respectively, for a nozzle temperature of 230 °C, printing speed of 55 mm/s, layer height of 0.2 mm and infill of 100%. Authors adopted these printing parameters without any optimization study and, given the results obtained, they were 16.3% and 11.8% lower than those verified in the present study. Therefore, it is possible to conclude that the optimization is important to maximize the mechanical properties, and, with this procedure, it is possible to obtain significant improvements. In another study, Walter et al. [54] used a printing speed of 50 mm/s, infill of 100%, raster angle of [+45/−45], layer height of 0.1 and 0.3 mm, and nozzle temperatures of 210, 230, 250 and 265 °C to produce PETG and PETG+CF samples. They observed that, while the bending modulus for neat PETG did not present significant changes for the temperatures studied, around 1.5 GPa and 1.9 GPa for a layer height of 0.3 mm and 0.1 mm, respectively, the values for PETG+CF were 160% higher and increased with increasing temperature. This increase was explained by the authors due to the degree of porosity. A similar trend was observed for the bending strength, and both properties (bending strength and modulus) were clearly higher when samples were printed at nozzle temperatures above 230 °C. For example, for the PETG+CF composite, and considering a layer height of 0.1 mm, the bending strength increased from 60 MPa for 210 °C to close to 100 MPa for 265 °C. However, for a layer height of 0.3 mm, these values were 42 MPa and close to 100 MPa, respectively. Note that the maximum value is higher than that obtained here, which can be explained by the higher weight content of carbon fibres in the filament used by these authors. In terms of neat PETG, bending strength presents values similar to those obtained in this study. Therefore, while for neat PETG the nozzle temperature had almost no influence on the bending properties and the layer height had only a marginal influence, for PETG+CF composites the nozzle temperature clearly influenced the properties. This influence was explained by the authors due to the lower porosity and the fact that the energy of the extruded polymer increases the local temperature of the previous layer, creating stronger bonds between layers and, consequently, better mechanical properties.

Evidence common to this study and those reported previously [53,54] is the fact that mechanical properties are maximized for an infill of 100%, which is even suggested by the Pareto charts (Figure 6, Figure 7 and Figure 8) as the most influential parameter. In fact, a higher infill density promotes less porosity and, consequently, stronger interfacial connections between adjacent deposited filaments [55]. On the other hand, a low infill density causes the filaments to be deposited further away from each other and the weak bonds that form between them promote lower mechanical strength. Moreover, samples printed with higher infill percentages have more material in their constitution, resulting in denser samples with higher bearing capacity [15].

Regarding the nozzle temperature, different effects can be observed for the materials analysed. While for the PETG and PETG+KF composite the higher temperature (265 °C) was the best, which confirms the results obtained in the literature, for the PETG+CF the lower temperature (195 °C) was preferable to maximize the mechanical properties, which somewhat contradicts the literature. However, these results agree with the manufacturer’s datasheet, where values between 230 and 250 °C are suggested for PETG, and between 240 and 260 °C as well as from200 to 240 °C are suggested, respectively, for the PETG+KF and PETG+CF. In fact, this parameter influences the fluidity of the extruded filament, its solidification characteristics and the consequent inter-bonding strength between the solidified layers [36]. According to Ding et al. [38], the density of the extruded materials increases gradually with temperature, which leads to a smaller number of voids due to the higher fluidity of the material. Consequently, the printed samples exhibit better compactness, interfacial bonding, and mechanical performance. Berreta et al. [39] revealed that higher viscosities lead to more complex depositions of the filament layer, for which the printing speed must be adjusted. Furthermore, Guessasma et al. [56] studied the nozzle temperature effect on the tensile strength of PETG and the results showed that the filament must be printed at a temperature above 230 °C for the material to be pasted onto the platform, and at 250 °C they obtained a porosity of around 2%. Therefore, this study reveals, from the Pareto charts (Figure 5), that the nozzle temperatures studied (from 235 °C to 265 °C) have almost no influence on the bending properties for neat PETG, which is in line with the studies developed by Walter et al. [54] and all temperatures are above 230 °C as suggested by Guessasma et al. [56]. In terms of composites, literature reports that carbon fibres are excellent thermal conductors [57], therefore, their introduction into a polymer matrix can degrade the polymer when the filament is printed at higher temperatures. In this case, a lower nozzle temperature increases the mechanical performance because the thermal degradation of the polymer is minimized [58,59]. This evidence agrees with what was observed by Walter et al. [54], where for PETG+CF composites the nozzle temperature clearly influences the properties and confirms the Pareto analysis that gives this parameter as the most influential in maximizing the bending modulus and strain, while for the bending strength it is the second one. On the other hand, due to the low thermal conductivity of aramid fibres [60], resulting from their polymeric base, the viscosity of the composite is expected to be high (similar to neat PETG) and, consequently, the nozzle temperature is also similar to neat PETG. According to the Pareto charts, this parameter manifests an even smaller influence than that observed for neat PETG. However, despite porosity being unavoidable in both materials (neat polymers and composites), an increase in composites is expected due to the agglomeration of nanoparticles in the molten filament during its passage through the printer nozzle before deposition [40,41]. To complement this analysis, the authors carried out a study on the thermal conductivity of the different materials and found average values around 0.2046, 0.2146 and 0.187 W/mK, for PETG, PETG+CF and PETG+KF, respectively. From these results, shown in Table 13, it is possible to observe that the thermal conductivity of PETG+CF is the highest, followed by PETG and PETG+KF. Compared to the neat PETG, when carbon fibres are added, the thermal conductivity increases around 5%, while a decrease of 9% is found when the aramid fibres are added. In fact, the addition of carbon fibres increased the thermal conductivity because carbon fibres are excellent thermal conductors [61,62], while aramid fibres promote lower thermal conductivity compared to the neat polymer due to their low thermal conductivity [63]. In many situations they are even used as an insulator [64,65].

Literature reports that nozzle temperature (consequently the viscosity) and printing speed are related and must be analysed together to optimize the solidification conditions [47]. Therefore, in terms of neat PETG and the PETG+KF composite, for the polymer chains to diffuse and crystallize, a lower printing speed and, as mentioned above, a higher nozzle temperature (265 °C) is inevitable due to the viscosity of these materials [24]. In this case, the printing speed indicated by the Taguchi method was 20 mm/s, a value lower than the maximum of 30 mm/s recommended by Wang et al. [24] for materials with higher viscosities. Consequently, the extruded material has enough time to weld with the surrounding printed component, which improves the mechanical performance of the produced parts. On the other hand, for the PETG+CF composite, the highest printing speed (60 mm/s) is indicated as the preferable one because the excellent thermal conductivity of the carbon fibres promotes a lower viscosity of the composite. In this case, a higher printing speed reduces the volume of the extruded material, which is favourable for the lowest nozzle temperature suggested (195 °C). Despite the high printing speed, the printed layers overlap the previous ones that are not yet completely solidified, and a good adhesion is achieved between them. Therefore, it is possible to conclude that temperature and time (indirectly printing speed) have a significant effect on bond formation [49]. Furthermore, for all materials, the Pareto charts reveal that this parameter has the least influence, among those studied, on the mechanical properties.

Finally, in terms of layer height (layer thickness), very similar values were obtained for neat PETG and PETG+KF. While for the first material the optimal value was 0.4 mm, for the composite it was 0.35 mm. In fact, smaller layer thicknesses promote higher mechanical properties due to lower interlayer gaps and, consequently, fewer air pores in the cross-section [26]. According to Kovan et al. [27], the gaps between filament rasters have a strong influence on the adhesion properties and, in this context, a compressed filament deposition by the nozzle under adequate extrusion force can lead to a decrease in these gaps and a consequent increase in the inter-bonding strength. According to Sousa et al. [66], lower layer heights lead to higher mechanical properties due to higher cohesion between layers. Simultaneously, to achieve the same specimen size, more material will be necessary when thinner layers are used and, consequently, less porosity between layers due to their flattening. Therefore, more secondary bonds are formed, resulting in an overall increase in cohesive strength. Furthermore, to improve the mechanical properties, Gomez-Gras et al. [67] suggest that the nozzle diameter should be at least 1.5 times the layer thickness value to ensure strong cohesion between the printed filaments, which is in agreement with the values reported in this study for these materials. Finally, the lower layer height for the PETG+KF composite, in relation to neat PETG, is explained by the fact that the printed layers with higher thickness overlap the previous ones already solidified and, in this case, good adhesion is not guaranteed between them. On the other hand, for the PETG+CF composite, the layer thickness was the highest (0.53 mm), which may be related to the excellent thermal conductivity of the carbon fibres. Thicker layers promote a smaller number of layers, with a consequent decrease in the number of heating/cooling cycles, which can be favourable to minimize the thermal degradation caused by the local remelting of the previously solidified material necessary to bond the filaments [68]. Finally, according to Khan et al. [69], layer thickness is one of the main printing parameters that affects the elastic performance of the printed parts, which can be mainly proven by the Pareto analysis for both composites.

## 6. Conclusions

Fused filament fabrication (FFF) is the most popular additive manufacturing method, but it inevitably has some weaknesses. Probably the most important is the low mechanical properties of the parts obtained by this technique. This weakness can be overcome by resorting to post-treatment procedures, adding different types of reinforcements, and optimizing the printing parameters. However, due to the complex interaction between parameters, they must be analysed together (in a group). In terms of material, PETG (Poly(ethylene terephthalate)-glycol)) is one of the most used thermoplastics in 3D printing, replacing even the most traditional ones, arousing interest for numerous industrial applications that can be extended even further when reinforced with fibres.

Therefore, in this context, the present study intended to optimize the printing parameters to maximize the mechanical properties of neat PETG and reinforced with carbon fibres (PETG+CF) and aramid fibres (PETG+KF). For this purpose, the Taguchi and ANOVA analysis were used to minimize the number of parameter combinations and streamline the experimental procedure. Four factors were selected (nozzle temperature, printing speed, layer thickness and fill density values) and the theoretical results were compared with the experimental ones. Three-point bending tests were used to benefit from its sensitivity.

It was possible to conclude that the printing parameters that maximized the bending properties for neat PETG were a nozzle temperature of 265 °C, speed of 20 mm/s, layer height of 0.40 mm and an infill of 100%. Similar parameters were obtained for the PETG+KF composite, except for the layer height, which showed values of 0.35 mm. This was explained by the fact that printed layers with higher thicknesses, when overlapping the previous ones already solidified, do not guarantee a good adhesion between them. Regarding the PETG+CF composite, the values obtained were a nozzle temperature of 195 °C, speed of 60 mm/s, layer height of 0.53 mm and infill of 100%. These results, contrary to the previous ones, are a consequence of the thermal conductivity of the carbon fibres. In this case, higher thicknesses promote a smaller number of layers and, consequently, the smaller number of heating/cooling cycles minimizes the thermal degradation caused by the remelting of the previously solidified material necessary to join the filaments.

For these values, the experimental results led to a bending stress of 66.9 MPa for neat PETG, 79.2 MPa for the PETG+CF composite and 47.7 MPa for the PETG+KF composite. In terms of the bending modulus, they were 1.7 GPa, 3.6 GPa and 1.5 GPa, and for the bending strain they were 5.7%, 3.2% and 5.1%, respectively.

Another piece of evidence from this study reveals that temperature proved to be a very important parameter because it influences the fluidity of the extruded filament, its solidification characteristics and consequent inter-bonding strength between layers. Therefore, when carbon fibres are added to the polymer, a high temperature is not required due to its excellent thermal conductivity. Even higher temperatures are inadvisable because they can degrade the polymer. On the other hand, when aramid fibres are added to the polymer, higher temperatures are required due to the low thermal conductivity of the fibres. Complementary studies also showed that, compared to pure PETG, when carbon fibres were added, the thermal conductivity of the composite increased by around 5%, while when using aramid fibres, there was a decrease of 9%. Therefore, nozzle temperature and print speed must be related and analysed together to optimize the solidification conditions, especially for composites.

## Figures and Tables

**Table 1:** Controllable factors and associated levels used in Taguchi experimental design for PETG.

Factor	Factor Code	Level 1	Level 2	Level 3	Level 4	Level 5
Nozzle Temperature (°C)	A	225	235	245	255	265
Speed (mm/s)	B	20	30	40	50	60
Layer height (mm)	C	0.2	0.25	0.3	0.35	0.4
Infill (%)	D	20	40	60	80	100

**Table 2:** Controllable factors and associated levels used in Taguchi experimental design for PETG+CF.

Factor	Factor Code	Level 1	Level 2	Level 3	Level 4	Level 5
Nozzle Temperature (°C)	A	195	205	215	235	245
Speed (mm/s)	B	20	30	40	50	60
Layer height (mm)	C	0.35	0.4	0.45	0.5	0.55
Infill (%)	D	20	40	60	80	100

**Table 3:** Controllable factors and associated levels used in Taguchi experimental design for PETG+KF.

Factor	Factor Code	Level 1	Level 2	Level 3	Level 4	Level 5
Nozzle Temperature (°C)	A	235	245	255	260	265
Speed (mm/s)	B	20	30	40	50	60
Layer height (mm)	C	0.35	0.4	0.45	0.5	0.55
Infill (%)	D	20	40	60	80	100

**Table 4:** Bending experimental results detailed obtained using Taguchi L16 (4^5^) for PETG.

Run	A	B	C	D	Bending Stress (MPa)	Bending Modulus (GPa)	Bending Strain (%)
1	225	20	0.2	20	30.75 ± 1.44	1.04 ± 0.07	3.64 ± 0.50
2	225	30	0.25	40	37.05 ± 3.64	1.13 ± 0.07	4.11 ± 0.48
3	225	40	0.3	60	46.95 ± 1.67	1.33 ± 0.07	4.76 ± 0.09
4	225	50	0.35	80	55.04 ± 2.34	1.30 ± 0.09	5.04 ± 0.20
5	225	60	0.4	100	63.14 ± 2.31	1.66 ± 0.11	5.47 ± 0.18
6	235	20	0.25	60	49.62 ± 1.87	1.38 ± 0.04	4.97 ± 0.09
7	235	30	0.3	80	55.10 ± 2.38	1.52 ± 0.05	5.52 ± 0.06
8	235	40	0.35	100	61.11 ± 4.02	1.64 ± 0.09	5.70 ± 0.06
9	235	50	0.4	20	35.74 ± 2.27	1.17 ± 0.05	4.02 ± 0.44
10	235	60	0.2	40	35.91 ± 1.29	1.12 ± 0.11	4.04 ± 0.35
11	245	20	0.3	100	66.66 ± 1.99	1.71 ± 0.08	5.55 ± 0.12
12	245	30	0.35	20	38.60 ± 4.02	1.20 ± 0.05	4.08 ± 0.09
13	245	40	0.4	40	46.70 ± 2.41	1.35 ± 0.11	4.42 ± 0.45
14	245	50	0.2	60	46.38 ± 1.33	1.31 ± 0.43	4.64 ± 0.05
15	245	60	0.25	80	54.48 ± 1.71	1.45 ± 0.06	5.10 ± 0.11
16	255	20	0.35	40	49.38 ± 2.42	1.39 ± 0.12	4.68 ± 0.13
17	255	30	0.4	60	57.47 ± 1.90	1.54 ± 0.11	5.18 ± 0.25
18	255	40	0.2	80	57.16 ± 1.39	1.49 ± 0.07	5.46 ± 0.17
19	255	50	0.25	100	65.05 ± 1.56	1.62 ± 0.09	5.76 ± 0.10
20	255	60	0.3	20	35.36 ± 1.09	1.13 ± 0.03	3.98 ± 0.23
21	265	20	0.4	80	53.56 ± 2.83	1.36 ± 0.09	5.61 ± 0.12
22	265	30	0.2	100	67.07 ± 1.43	1.69 ± 0.06	5.44 ± 0.26
23	265	40	0.25	20	33.62 ± 0.65	1.17 ± 0.02	4.09 ± 0.21
24	265	50	0.3	40	42.83 ± 1.52	1.26 ± 0.06	4.64 ± 0.19
25	265	60	0.35	60	52.59 ± 2.46	1.42 ± 0.09	5.22 ± 0.14

**Table 5:** Bending experimental results detailed obtained using Taguchi L16 (4^5^) for PETG+CF.

Run	A	B	C	D	Bending Stress (MPa)	Bending Modulus (GPa)	Bending Strain (%)
1	195	20	0.35	20	61.31 ± 3.48	2.94 ± 0.50	4.08 ± 0.94
2	195	30	0.4	40	64.53 ± 6.34	3.07 ± 0.32	4.79 ± 0.14
3	195	40	0.45	60	68.57 ± 1.79	2.67 ± 1.20	5.29 ± 0.04
4	195	50	0.5	80	71.99 ± 4.42	3.52 ± 0.24	5.89 ± 0.29
5	195	60	0.55	100	60.12 ± 2.32	2.80 ± 0.17	6.83 ± 0.31
6	205	20	0.4	60	64.13 ± 6.61	3.11 ± 0.31	4.98 ± 0.21
7	205	30	0.45	80	72.75 ± 2.53	3.17 ± 0.28	6.12 ± 0.12
8	205	40	0.5	100	69.76 ± 1.40	2.95 ± 0.12	5.91 ± 0.07
9	205	50	0.55	20	50.32 ± 2.42	2.18 ± 0.16	5.61 ± 0.08
10	205	60	0.35	40	63.63 ± 1.65	3.21 ± 0.09	4.71 ± 0.04
11	215	20	0.45	100	68.13 ± 4.02	3.03 ± 0.15	6.26 ± 0.04
12	215	30	0.5	20	49.63 ± 0.92	2.24 ± 0.07	4.93 ± 0.10
13	215	40	0.55	40	50.69 ± 0.82	2.19 ± 0.07	5.60 ± 0.21
14	215	50	0.35	60	63.22 ± 1.45	2.97 ± 0.11	4.99 ± 0.07
15	215	60	0.4	80	66.36 ± 1.15	2.89 ± 0.09	5.66 ± 0.04
16	235	20	0.5	40	44.17 ± 1.81	1.81 ± 0.18	5.30 ± 0.19
17	235	30	0.55	60	43.15 ± 1.67	1.66 ± 0.06	6.12 ± 0.09
18	235	40	0.35	80	58.74 ± 1.20	2.47 ± 0.06	5.70 ± 0.11
19	235	50	0.4	100	59.23 ± 1.22	2.40 ± 0.06	5.91 ± 0.12
20	235	60	0.45	20	40.60 ± 0.66	1.80 ± 0.11	5.24 ± 0.06
21	245	20	0.55	80	42.17 ± 1.79	1.54 ± 0.11	6.40 ± 0.15
22	245	30	0.35	100	64.58 ± 1.94	2.60 ± 0.14	6.16 ± 0.13
23	245	40	0.4	20	43.28 ± 2.31	1.92 ± 0.18	5.08 ± 0.18
24	245	50	0.45	40	46.39 ± 1.60	1.96 ± 0.08	5.59 ± 0.21
25	245	60	0.5	60	45.19 ± 1.69	1.70 ± 0.09	6.17 ± 0.19

**Table 6:** Bending experimental results detailed obtained using Taguchi L16 (4^5^) for PETG+KF.

Run	A	B	C	D	Bending Stress (MPa)	Bending Modulus (GPa)	Bending Strain (%)
1	235	20	0.35	20	30.91 ± 1.40	1.12 ± 0.11	7.02 ± 0.18
2	235	30	0.4	40	35.43 ± 0.92	1.22 ± 0.06	7.11 ± 0.12
3	235	40	0.45	60	39.24 ± 1.18	1.28 ± 0.07	7.83 ± 0.29
4	235	50	0.5	80	41.73 ± 1.03	1.29 ± 0.06	8.47 ± 0.22
5	235	60	0.55	100	39.82 ± 1.74	1.22 ± 0.09	8.86 ± 0.19
6	245	20	0.4	60	35.73 ± 1.06	1.25 ± 0.05	7.62 ± 0.17
7	245	30	0.45	80	38.81 ± 1.64	1.25 ± 0.06	8.86 ± 0.06
8	245	40	0.5	100	41.62 ± 5.77	1.33 ± 0.07	8.97 ± 0.17
9	245	50	0.55	20	30.33 ± 1.04	1.02 ± 0.05	7.42 ± 0.21
10	245	60	0.35	40	33.58 ± 0.40	1.21 ± 0.03	6.94 ± 0.08
11	255	20	0.45	100	44.65 ± 2.18	1.36 ± 0.10	9.08 ± 0.27
12	255	30	0.5	20	32.02 ± 0.95	1.12 ± 0.05	6.81 ± 0.21
13	255	40	0.55	40	34.28 ± 2.18	1.10 ± 0.08	7.91 ± 0.15
14	255	50	0.35	60	37.22 ± 0.78	1.25 ± 0.03	7.76 ± 0.13
15	255	60	0.4	80	39.62 ± 0.64	1.28 ± 0.03	8.55 ± 0.14
16	260	20	0.5	40	38.14 ± 0.70	1.15 ± 0.04	7.94 ± 0.17
17	260	30	0.55	60	38.06 ± 1.54	1.16 ± 0.07	8.52 ± 0.10
18	260	40	0.35	80	42.09 ± 1.05	1.36 ± 0.06	8.50 ± 0.27
19	260	50	0.4	100	40. 50 ± 1.42	1.26 ± 0.08	8.56 ± 0.19
20	260	60	0.45	20	29.20 ± 1.23	1.02 ± 0.07	7.55 ± 0.17
21	265	20	0.55	80	41.09 ± 2.67	1.22 ± 0.12	9.18 ± 0.21
22	265	30	0.35	100	47.35 ± 2.03	1.50 ± 0.07	9.12 ± 0.16
23	265	40	0.4	20	31.31 ± 0.95	1.15 ± 0.03	6.96 ± 0.09
24	265	50	0.45	40	35.02 ± 0.87	1.12 ± 0.06	7.77 ± 0.16
25	265	60	0.5	60	37.91 ± 0.80	1.15 ± 0.03	8.36 ± 0.15

**Table 7:** ANOVA analysis for PETG.

Source	DF	Adj SS	Adj MS	F-Value	*p*-Value	Remarks
Bending stress						
Regression	4	2761.19	690.30	86.65	<0.001	Significant
Nozzle Temp. (°C)	1	73.00	73.00	9.16	0.007	Significant
Speed (mm/s)	1	14.80	14.80	1.86	0.188	Insignificant
Layer height (mm)	1	61.79	61.79	7.76	0.011	Significant
Infill (%)	1	2611.61	2611.61	327.84	<0.001	Significant
Error	20	159.32	7.97			
Total	24	2920.52				
Bending modulus						
Regression	4	0.806934	0.201734	30.92	<0.001	Significant
Nozzle Temp. (°C)	1	0.030027	0.030027	4.60	0.044	Significant
Speed (mm/s)	1	0.007923	0.007923	1.21	0.284	Insignificant
Layer height (mm)	1	0.021448	0.021448	3.29	0.085	Insignificant
Infill (%)	1	0.747536	0.747536	114.59	<0.001	Significant
Error	20	0.130475	0.006524			
Total	24	0.937410				
Bending strain						
Regression	4	9.6222	2.40556	98.94	<0.001	Significant
Nozzle Temp. (°C)	1	0.4574	0.45737	18.81	<0.001	Significant
Speed (mm/s)	1	0.0446	0.04464	1.84	0.191	Insignificant
Layer height (mm)	1	0.2659	0.26588	10.94	0.004	Significant
Infill (%)	1	8.8543	8.85434	364.16	<0.001	Significant
Error	20	0.4863	0.02431			
Total	24	10.1085				
Regression	4	2761.19	690.30	86.65	<0.001	Significant

**Table 8:** ANOVA analysis for PETG+CF.

Source	DF	Adj SS	Adj MS	F-Value	*p*-Value	Remarks
Bending stress						
Regression	4	2297.50	574.38	46.16	<0.001	Significant
Nozzle Temp. (°C)	1	782.46	782.46	62.88	<0.001	Significant
Speed (mm/s)	1	9.96	9.96	0.80	0.382	Insignificant
Layer height (mm)	1	182.68	182.68	14.68	0.001	Significant
Infill (%)	1	1322.40	1322.40	106.27	<0.001	Significant
Error	20	248.87	12.44			
Total	24	2546.37				
Bending modulus						
Regression	4	6.51456	1.62864	43.49	<0.001	Significant
Nozzle Temp. (°C)	1	4.03117	4.03117	107.64	<0.001	Significant
Speed (mm/s)	1	0.00585	0.00585	0.16	0.697	Insignificant
Layer height (mm)	1	0.85626	0.85626	22.86	<0.001	Significant
Infill (%)	1	1.62128	1.62128	43.29	<0.001	Significant
Error	20	0.74904	0.03745			
Total	24	7.26360				
Bending strain						
Regression	4	4.17841	1.04460	66.78	<0.001	Significant
Nozzle Temp. (°C)	1	1.73389	1.73389	110.84	<0.001	Significant
Speed (mm/s)	1	0.00031	0.00031	0.02	0.889	Insignificant
Layer height (mm)	1	1.26760	1.26760	81.03	<0.001	Significant
Infill (%)	1	1.17661	1.17661	75.22	<0.001	Significant
Error	20	0.31286	0.01564			
Total	24	4.49127				
Regression	4	2297.50	574.38	46.16	<0.001	Significant

**Table 9:** ANOVA analysis for PETG+KF.

Source	DF	Adj SS	Adj MS	F-Value	*p*-Value	Remarks
Bending stress						
Regression	4	342.150	85.537	66.41	<0.001	Significant
Nozzle Temp. (°C)	1	0.003	0.003	0.00	0.964	Insignificant
Speed (mm/s)	1	20.084	20.084	15.59	0.001	Significant
Layer height (mm)	1	0.029	0.029	0.02	0.882	Insignificant
Infill (%)	1	322.034	322.034	250.03	<0.001	Significant
Error	20	25.759	1.288			
Total	24	367.909				
Bending modulus						
Regression	4	0.154184	0.038546	92.99	<0.001	Significant
Nozzle Temp. (°C)	1	0.001158	0.001158	2.79	0.110	Insignificant
Speed (mm/s)	1	0.020467	0.020467	49.37	<0.001	Significant
Layer height (mm)	1	0.029268	0.029268	70.61	<0.001	Significant
Infill (%)	1	0.103291	0.103291	249.18	<0.001	Significant
Error	20	0.008291	0.000415			
Total	24	0.162474				
Bending strain						
Regression	4	5.22057	1.30514	39.98	<0.001	Significant
Nozzle Temp. (°C)	1	0.00668	0.00668	0.20	0.656	Insignificant
Speed (mm/s)	1	0.09363	0.09363	2.87	0.106	Insignificant
Layer height (mm)	1	0.35059	0.35059	10.74	0.004	Significant
Infill (%)	1	4.76967	4.76967	146.11	<0.001	Significant
Error	20	0.65290	0.03265			
Total	24	5.87347				

**Table 10:** Model summary for all materials.

Response	S	R-sq	R-sq(adj)	R-sq(pred)
PETG				
Bending stress	2.82244	94.54%	93.45%	90.29%
Bending modulus	0.0807698	86.08%	83.30%	74.73%
Bending strain	0.155930	95.19%	94.23%	92.35%
PETG+CF				
Bending stress	3.52752	90.23%	88.27%	82.69%
Bending modulus	0.193525	89.69%	87.63%	82.88%
Bending strain	0.125072	93.03%	91.64%	89.42%
PETG+KF				
Bending stress	1.13488	93.00%	91.60%	88.94%
Bending strain	0.0203600	94.90%	93.88%	91.51%
Bending modulus	0.180679	88.88%	86.66%	82.15%

**Table 11:** Optimal 10 solutions obtained by desirability function for all materials.

Solution	Nozzle Temperature (°C)	Speed (mm/s)	Layer Height (mm)	Infill (%)	Bending Stress (MPa) Fit	Bending Modulus (GPa) Fit	Bending Strain (%) Fit	Composite Desirability
PETG								
1	265.000	20.0000	0.400000	100.000	69.6761	1.73539	6.08315	0.99998
2	265.000	60.0000	0.400000	100.000	67.4999	1.68504	5.96363	0.98519
3	265.000	20.0000	0.200000	100.000	65.2296	1.65255	5.79147	0.95179
4	265.000	20.0000	0.201306	97.386	64.3140	1.63710	5.73837	0.93227
5	225.000	20.0000	0.400000	100.000	64.8431	1.63737	5.70059	0.93162
6	225.000	20.0000	0.400000	100.000	64.8431	1.63737	5.70058	0.93162
7	225.000	20.0000	0.400000	100.000	64.8431	1.63737	5.70058	0.93162
8	265.000	20.0000	0.400000	83.658	63.7709	1.63548	5.73931	0.92651
9	225.001	59.9997	0.400000	100.000	62.6669	1.58701	5.58107	0.86758
10	265.000	20.0000	0.400000	43.127	49.1245	1.38769	4.88649	0.53551
PETG+CF								
1	195.000	60.0000	0.525121	100.000	72.7516	3.18991	3.64024	0.802270
2	195.000	60.0000	0.525121	100.000	72.7516	3.18991	3.64024	0.802270
3	195.000	60.0000	0.550000	100.000	71.8005	3.12479	3.71947	0.800580
4	195.000	60.0000	0.550000	100.000	71.8005	3.12479	3.71947	0.800580
5	195.000	59.9938	0.550000	99.972	71.7932	3.12454	3.71926	0.800424
6	195.000	20.0000	0.511487	100.000	71.4874	3.18233	3.60680	0.782423
7	195.011	20.0000	0.550000	100.000	70.0117	3.08129	3.72960	0.779197
8	195.015	20.0000	0.550000	100.000	70.0105	3.08120	3.72966	0.779181
9	195.002	20.0000	0.350000	100.000	77.6602	3.60494	3.09257	0.687411
10	195.002	20.0000	0.350000	100.000	77.6602	3.60494	3.09257	0.687411
PETG+KF								
1	265.000	20.0000	0.350000	100.000	42.8514	1.38203	5.07710	0.896739
2	265.000	20.0000	0.478304	100.000	42.7895	1.31995	5.29197	0.882550
3	265.000	20.0000	0.550000	100.000	42.7548	1.28526	5.41204	0.859197
4	235.001	20.0000	0.550000	100.000	42.7263	1.26630	5.36653	0.834593
5	264.998	59.6749	0.350000	99.816	40.3135	1.30134	5.24594	0.795999
6	265.000	59.9983	0.350000	99.687	40.2768	1.30040	5.24536	0.794019
7	265.000	59.9983	0.350000	99.683	40.2762	1.30039	5.24530	0.793985
8	265.000	59.9981	0.350000	98.450	40.1198	1.29759	5.22626	0.783726
9	265.000	25.2370	0.350000	70.783	38.8121	1.30504	4.64857	0.635495
10	264.990	56.8432	0.350000	59.987	35.4390	1.21655	4.61860	0.470369

**Table 12:** Comparison between experimental and theoretical results.

Materials.	Condition	Bending Stress (MPa)	Bending Modulus (GPa)	Bending Strain (%)
PETG	Experimental	66.9 ± 1.43	1.70 ± 0.025	5.66 ± 0.06
	Theoretical	69.68 (−3.95%)	1.74 (−2.3%)	6.08 (−6.91%)
PETG+CF	Experimental	79.2 ± 0.97	3.55 ± 0.18	3.23 ± 0.21
	Theoretical	72.75 (9.69%)	3.19 (11.29%)	3.64 (−11.26%)
PETG+KF	Experimental	47.7 ± 0.97	1.53 ± 0.27	5.10 ± 0.1
	Theoretical	42.85 (11.32%)	1.38 (10.87%)	5.08 (0.39%)

**Table 13:** Results of the thermal analysis for PETG, PETG+CF and PETG+KF.

Material	Thermal Conductivity (W/mK)
PETG	0.2046 ± 0.0007
PETG+CF	0.2146 ± 0.0004
PETG+KF	0.187 ± 10.0005

## Data Availability

Not applicable.
